# Stride Velocity 95^th^ Centile Detects Decline in Ambulatory Function Over Shorter Intervals than the 6-Minute Walk Test or North Star Ambulatory Assessment in Duchenne Muscular Dystrophy

**DOI:** 10.3233/JND-230188

**Published:** 2024-04-30

**Authors:** Michael Rabbia, Maitea Guridi Ormazabal, Hannah Staunton, Klaas Veenstra, Damien Eggenspieler, Mélanie Annoussamy, Laurent Servais, Paul Strijbos

**Affiliations:** a Genentech Inc., 1 DNA Way, South San Francisco, CA, USA; b F. Hoffmann-La Roche Ltd, Grenzacherstrasse 124, Basel, Switzerland; c Roche Products Ltd, Hexagon Place, Shire Park, 6 Falcon Way, Welwyn Garden City, UK; d SYSNAV, 72 Rue Emile Loubet, Vernon, France; e MDUK Oxford Neuromuscular Centre and NIHR Oxford Biomedical Research Centre, University of Oxford, Oxford, UK; f Department of Pediatrics, Division of Child Neurology, Centre de Référence des Maladies Neuromusculaires, University Hospital Liège and University of Liège, Avenue de l’Hôpital 1, Liege, Belgium

**Keywords:** muscular, dystrophy, digital endpoints, ambulation, stride velocity 95^th^ centile, clinical trials

## Abstract

**Background:**

Stride Velocity 95^th^ Centile (SV95C) is the first wearable device-derived clinical outcome assessment (COA) to receive European Medicines Agency (EMA) qualification as a primary endpoint in ambulant patients with Duchenne muscular dystrophy (DMD) aged ≥4 years.

**Objective:**

To compare SV95C—in its first-ever clinical trial application as a secondary endpoint—with established motor function COAs used in the trial (Four-Stair Climb [4SC] velocity, North Star Ambulatory Assessment [NSAA], and Six-Minute Walk Distance [6MWD]).

**Methods:**

SV95C was a secondary endpoint in a subset (*n* = 47) of participants in the SPITFIRE/WN40227 trial of taldefgrobep alfa, which was discontinued due to lack of clinical benefit. Participants in the ≤48-week SV95C sub-study were 6–11 years old and received corticosteroids for ≥6 months pre-treatment. Pearson correlations were used to compare SV95C with the other COAs. Responsiveness and changes over time were respectively assessed via standardized response means (SRMs) based on absolute changes and mixed models for repeated measures.

**Results:**

SV95C change at Week 24 was –0.07 m/s, with limited variability (standard deviation: 0.16, *n* = 27). The SRM for SV95C indicated moderate responsiveness to clinical change at the earliest timepoint (Week 12, *n* = 46), while those of the other COAs did not indicate moderate responsiveness until Week 36 (6MWD, *n* = 33) or Week 48 (4SC velocity, *n* = 20; NSAA total score, *n* = 20). Baseline correlations between SV95C and other COAs were strong (*r* = 0.611–0.695). Correlations between SV95C change from baseline to Week 48 and changes in other COAs were moderate to strong (*r* = 0.443–0.678).∥

**Conclusions:**

Overall, SV95C demonstrated sensitivity to ambulatory decline over short intervals, low variability, and correlation with established COAs. Although the negative trial precluded demonstration of SV95C’s sensitivity to drug effect, these findings support the continued use of SV95C in DMD clinical trials.

## INTRODUCTION

Duchenne muscular dystrophy (DMD) is an X-linked, recessive, degenerative neuromuscular disorder caused by mutations in the *DMD* gene, which encodes dystrophin protein [1]. The absence of dystrophin protein causes skeletal and cardiac muscle to degenerate, leading to progressive muscle weakness and wasting that is characteristic of the disease [1]. Although a number of different therapies for DMD have been studied, including dystrophin gene therapy, none of the available treatments to date stop disease progression [2]. Even with available glucocorticoid treatments, patients typically lose ambulation and are wheelchair-bound by the time they are 8 to 14 years old [3].

Despite significant investment and a decade of large-scale clinical development, there is currently no cure for DMD [4]. In this context, there is a need in DMD trials for improved endpoints that are objective, sensitive to therapeutic intervention, and reflective of real-world functionality—not only to avoid falsely negative Phase 3 trials but also to properly inform decision-making in the early phases of drug development, reduce patient burden, and avoid wasting resources in late-phase clinical trials [5].

Established motor function clinical outcome assessments (COAs) used to assess the efficacy of investigational therapies for DMD in clinical trials are typically clinician-administered, performance-based tasks that the patient completes during office visits. Several compounds assessed in preclinical and clinical studies of DMD have failed to complete clinical development [5]; some of these failures may be attributed to difficulties demonstrating a statistically significant difference in a currently available clinical endpoint over a reasonable period of time. The current primary outcomes in Phase 3 trials of DMD that are therapeutically focused on preserving ambulation are the 6-minute walk distance (6MWD) [6, 7] and the North Star Ambulatory Assessment (NSAA) [8]. As these assessments have been robustly developed and validated in patients with DMD [6–8], they have been used in several pivotal trials. However, none of these trials demonstrated a significant difference between treated and untreated groups, so these endpoints have not yet been used to support drug approval as primary outcomes in a clinical trial demonstrating drug efficacy (as few therapies have been approved for DMD). These COAs also have certain limitations. Firstly, they require evaluation in a clinical setting, which contributes to patient burden and limits the geographic breadth of clinical trials to within a commutable radius of the specialist centers that administer these assessments. Secondly, assessments conducted in a clinical setting are scheduled at specific timepoints and could therefore be influenced by patient condition and motivation at a given visit. Wearable devices, remote sensors, and other digital tools that enable continuous and sensitive measurement of functional ability in real-world settings may help to circumvent these limitations.

Stride Velocity 95^th^ Centile (SV95C) is a digital measure of peak ambulation performance that represents the minimum velocity of the top 5% most rapid strides taken by a wearer in a real-world setting [9]. In other words, 95% of strides are slower than SV95C and 5% are faster. SV95C (measured at the ankle by a valid and suitable wearable device) is the first wearable acquired digital endpoint to receive European Medicines Agency (EMA) qualification for use as a primary endpoint to quantify the ambulatory ability of patients with DMD aged 4 years and older [10, 11], and is also currently under review by the US Food and Drug Administration as part of the COA qualification pathway [12].

SV95C can be measured by any wearable device worn at the ankle that meets the precision requirements outlined within the EMA guidance document on the clinical investigation of medicinal products for the treatment of DMD and Becker muscular dystrophy [13]. These specified requirements far exceed the precision levels of mainstream actimeters [14] and only one device—a Class I wearable medical device using precision magneto-inertial sensors to measure acceleration, velocity, and angular movements—meets this standard so far [9, 14]. The device consists of two strap-based wearable sensors and a base station for device charging and data transfer. Outcomes derived from this wearable are currently used in studies of several diseases, such as DMD, spinal muscular atrophy, facioscapulohumeral muscular dystrophy, and limb girdle muscular dystrophy [15, 16]. Additionally, normative values have been established [17]. This wearable magneto-inertial technology allows data to be collected continuously with high accuracy and precision over prolonged time periods in uncontrolled environments during normal daily living [14].

SV95C, measured by ActiMyo^®^, a device that meets EMA precision recommendations, was included as a secondary endpoint to evaluate the efficacy of taldefgrobep alfa (RG6206, talditercept alfa, RO7239361, previously BMS-986089) compared with placebo in the SPITFIRE/WN40227 clinical trial (NCT03039686) in ambulatory patients with DMD. This was the first Phase 3 clinical trial in patients with DMD to incorporate SV95C as a secondary endpoint.

In this article, we share insights from the novel application of SV95C in a clinical trial assessing taldefgrobep alfa in patients with DMD. The trial was discontinued due to futility.

## MATERIALS AND METHODS

### Clinical trial details

SPITFIRE/WN40227 (NCT03039686) was a Phase 2/3, randomized, placebo-controlled study to assess the efficacy, safety, and tolerability of taldefgrobep alfa in ambulatory boys with DMD aged ≥6 to < 12 years at randomization [18]. Participants were required to have been receiving corticosteroids (prednisone, prednisolone, or deflazacort) for at least 6 months before administration of the study drug, with no significant corticosteroid dose or regimen changes for at least 3 months pre-treatment and the regimen expected to remain stable throughout the 48-week double-blind phase of the study. Patients were randomized 1:1:1 to receive either high-dose taldefgrobep alfa, low-dose taldefgrobep alfa, or placebo. The low dose was 7.5 mg for patients weighing ≤40 kg and 15 mg for patients > 40 kg. The high dose was 35 mg for patients weighing ≤40 kg and 50 mg for patients > 40 kg. After completing the 48-week double-blind period of the study, patients could enter an open-label extension period (≤192 weeks), in which all patients received taldefgrobep alfa.

SV95C was measured using the ActiMyo^®^ device (SYSNAV, Vernon, France), which consists of two magneto-inertial wearable sensors that continuously capture a patient’s movement during a maximum period of 16 hours. Data are uploaded at night when the wearable is placed on an internet-connected docking station [19].

Use of the wearable device was introduced as an optional assessment and SV95C was prespecified as a secondary endpoint through a protocol amendment dated 16 August 2018, approximately 13 months after the first patient was randomized (the first patient was randomized on 6 July 2017 and the last patient’s last visit was 28 April 2020). As the study was already underway when the protocol was amended to introduce the wearable device and SV95C, and the protocol required the collection of pre-treatment baseline data during the screening/baseline period, only newly recruited patients at selected sites were offered the opportunity to participate. Therefore, only a subset (*N* = 52) of the overall SPITFIRE patient population was included in the sub-study.

During the screening visit, the investigator explained the wearable device to the patients and their families and taught them how to use the device. Each patient who agreed to use the device received a briefcase to take home that included the wearable sensors and docking station, power and internet cables, an instruction manual, a quick start guide, and a patient-friendly flier with facts about the device. Patients were asked to wear the device every day during all activities, except when showering or sleeping, for 6-week periods every 3 months (in between clinic visits), as well as during the clinic visits. To ensure consistent and sustained use of the wearable device by patients, the clinical team monitored and responded to the compliance score for each patient. Stride identification and quantification were performed on aggregated periods and no action was made to identify or individualize specific activity outside of walking and running episodes and climbing/descending stairs.

The SPITFIRE study (and the development of taldefgrobep alfa) was discontinued following the outcome of a pre-planned futility analysis of the primary endpoint and selected secondary endpoints, which was conducted after approximately 30% of the patients had completed 48 weeks of the study drug treatment [18]. The futility analysis suggested that treatment with taldefgrobep alfa was unlikely to translate into meaningful functional change, and the study had a very low likelihood of meeting the primary endpoint of demonstrating a treatment difference in change from baseline to Week 48 in the NSAA total score. Due to the early termination, most patients had less than 48 weeks of data collected with the wearable device.

### Ethics

SPITFIRE/WN40227 was conducted in accordance with the Helsinki Declaration of 1964 and its amendments, and with Good Clinical Practice guidelines. Institutional Review Board/Independent Ethics Committee approval was obtained at each study site prior to study initiation, and studies were registered with ClinicalTrials.gov. Participants (or, in the case of minors, parents, guardians, or legally acceptable representatives) signed written informed consent prior to study participation.

### Statistical analyses

Here we report the outcomes of exploratory *post-hoc* analyses of SV95C in the SPITFIRE/WN40227 clinical trial population, collected in a sub-study using the wearable device (the ‘SV95C evaluable population’). The SV95C evaluable population includes all patients who agreed to wear the device to collect daily ambulatory data, had a baseline SV95C measurement, and had at least one post-baseline SV95C measurement (≥50 hours of recording) (*N* = 47). SV95C recordings were aggregated, and all data collected during the recording period were analyzed.

Demographics and baseline characteristics are summarized by treatment received during SPITFIRE/WN40227 (placebo [*n* = 15]; any taldefgrobep alfa dose [*n* = 32]) as well as across all patients (*N* = 47).

Values and absolute change from baseline values for SV95C in meters per second (m/s), Four-Stair Climb (4SC) velocity in stairs per second, NSAA total score, and 6MWD in meters are summarized at each visit during the double-blind treatment period for the SV95C evaluable population (Baseline, Week 12, Week 24, Week 36, and Week 48).

Standard scoring procedures were used for 4SC velocity, NSAA total score, and 6MWD. Briefly, 4SC velocity is scored by counting the number of stairs climbed per second (dividing the recorded 4SC time by four) [20]. The NSAA consists of 17 items that are scored from 0–2 depending on a patient’s ability to complete the activity; the total score ranges from 0–34, with higher scores indicating greater motor functional ability [21]. The 6MWD is scored by measuring the distance in meters walked by a patient in 6 minutes [22].

Standardized response means (SRMs)—indices of effect size used to estimate the responsiveness of a measure to clinical change [23]—were calculated for each measure (SV95C, 4SC velocity, NSAA total score, and 6MWD) at Weeks 12, 24, 36, and 48 for the overall population. SRMs were calculated by dividing the mean score change from baseline by the standard deviation of the change. SRMs of ≥0.20–0.50, ≥0.50– <0.80, and ≥0.80 purportedly represent small, moderate, and large responsiveness, respectively [24].

Least squares mean (LSM) change from baseline in outcome measures was assessed via Mixed Model for Repeated Measures (MMRM) analyses. LSMs are averages or predictions from a linear model that are used to summarize the effects of factors and test linear contrasts among predictions in experimental data [25]. Separate analyses were conducted for SV95C, 6MWD, 4SC velocity, and NSAA. Fixed effects included in the model were treatment, visit, and treatment-by-visit interaction. Baseline score on the outcome measure (SV95C, 6MWD, 4SC velocity, or NSAA) was included as a covariate. Patient ID was included in the model as a random effect.

Correlational analyses included baseline correlations between SV95C and 4SC velocity, NSAA, or 6MWD, as well as correlations between change from baseline in SV95C and change from baseline in 4SC velocity, NSAA, or 6MWD at Week 48. Correlations were considered very strong at correlation coefficients of 0.80–1.00, strong at 0.60–0.79, moderate at 0.40–0.59, weak at 0.20–0.39, and very weak at 0.00–0.19 [26]. Associations between SV95C and 4SC velocity, NSAA, or 6MWD were plotted as regression lines with confidence bands representing the 95% confidence interval of the predicted mean values.

## RESULTS

### 3.1 Patients

A total of 52 patients aged 6 to 11 years enrolled in the sub-study. Five of these patients were excluded from analyses because they did not have ≥50 recorded SV95C hours [10] at both baseline and at least one post-baseline timepoint. The remaining 47 patients constituted the SV95C evaluable population. Within the SV95C evaluable population, 32 patients received treatment with taldefgrobep alfa and 15 received placebo (Table 1).

**Table 1:** Baseline demographics in the SV95C evaluable population

	Placebo	Taldefgrobep alfa	Total
		any dose^*^
Age, years
*n*	15	32	47
Mean (SD)	7.8 (1.5)	8.7 (1.6)	8.4 (1.6)
Median (min–max)	8.0 (6–11)	9.0 (6–11)	8.0 (6–11)
Weight, kg
*n*	15	32	47
Mean (SD)	27.47 (7.34)	28.18 (8.76)	27.96 (8.26)
Median (min–max)	25.8 (19.5–44.7)	25.6 (15.2–50.9)	25.7 (15.2–50.9)
SV95C
*n*	15	32	47
Mean (SD)	1.66 (0.27)	1.56 (0.36)	1.59 (0.34)
Median (min–max)	1.64 (1.1–2.1)	1.52 (0.8–2.2)	1.60 (0.8–2.2)
NSAA total score
*n*	15	32	47
Mean (SD)	23.8 (5.3)	21.9 (6.3)	22.5 (6.0)
Median (min–max)	23.0 (15–33)	22.0 (8–33)	22.0 (8–33)
4SC velocity, stairs/s
*n*	15	32	47
Mean (SD)	1.23 (0.35)	1.15 (0.44)	1.18 (0.41)
Median (min–max)	1.21 (0.6–2.0)	1.13 (0.5–2.5)	1.18 (0.5–2.5)
6MWD, m
*n*	15	31	46
Mean (SD)	410.6 (55.4)	378.1 (65.6)	388.7 (63.7)
Median (min–max)	389.0 (347.0–510.3)	383.0 (173.0–467.0)	386.0 (173.0–510.3)

^*^Patients in SPITFIRE/WN40227 were treated with low-dose (7.5 mg for patients weighing≤40 kg and 15 mg for patients > 40 kg) or high-dose (35 mg for patients weighing≤40 kg and 50 mg for patients > 40 kg) taldefgrobep alfa. Due to the small sample sizes and overlap between the low- and high-dose panels, taldefgrobep alfa-treated patients were combined into a single treatment group. 4SC, Four-Stair Climb; 6WMD, Six-Minute Walk Distance; max, maximum; min, minimum; NSAA, North Star Ambulatory Assessment; SD, standard deviation; SV95C, Stride Velocity 95^th^ Centile.

Compliance information is presented in Supplementary Figure 1. At baseline, 51/52 patients (98.1%) had ≥50 hours of SV95C data and one patient (1.9%) had < 50 hours. At Week 12, 46 patients (88.5%) had ≥50 hours of SV95C measurement, two patients (3.8%) with data had < 50 hours, and four patients (7.7%) had no SV95C data. At Week 24, 27 patients (51.9%) had ≥50 hours of SV95C recordings, four (7.7%) had < 50 hours of data, and 21 (40.4%) had no SV95C data. At Week 36, early study termination resulted in a lack of SV95C data for 18 patients. Of the remaining 34 patients who had the opportunity to participate, 28 (82.4%) had ≥50 hours of SV95C data, one patient (2.9%) with data had < 50 hours, and five patients (14.7%) had no data. At Week 48, early study termination led to an absence of SV95C data for 33 patients. Of the remaining 19 patients who were able to continue participating, 16 (84.2%) had ≥50 hours of SV95C data and three (15.8%) had no data.

Baseline demographics were similar among patients treated with placebo and taldefgrobep alfa (Table 1). As there was no difference in outcomes between treatment groups in the SPITFIRE study [18], data from all 47 patients were pooled to form one population for analysis. The mean age of patients in the SV95C population at baseline was 8.4 years. At baseline, patients had a mean SV95C of 1.59 meters per second, a mean NSAA total score of 22.5, a mean 4SC velocity of 1.18 stairs per second, and a mean 6MWD of 388.7 meters.

### Correlations between SV95C and other motor function COAs

**Fig. 1:**
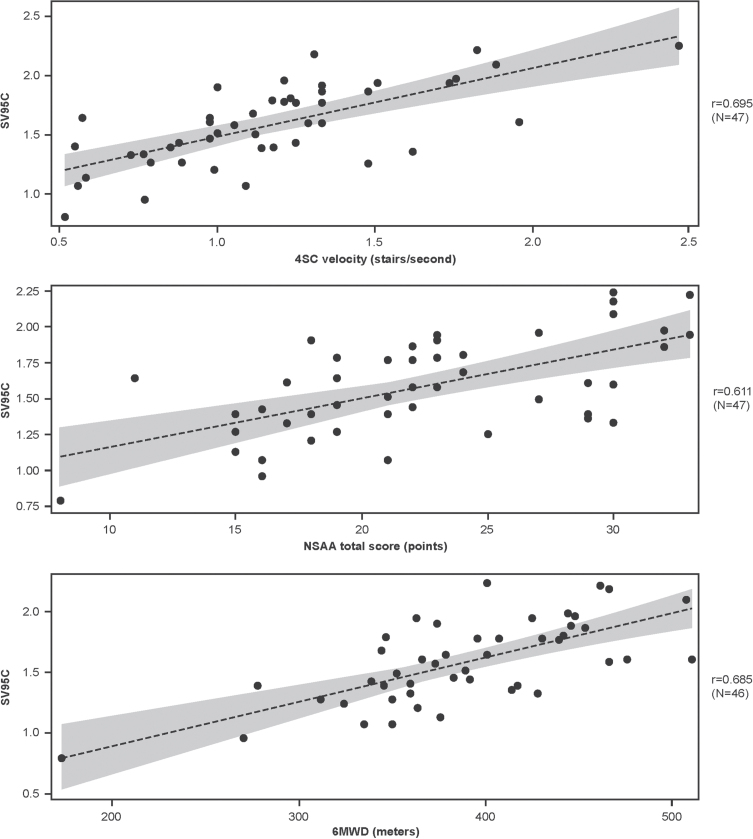
Associations between SV95C and other motor function COAs at baseline (regression lines with 95% confidence intervals for the predicted mean values). Pearson correlations. N’s reflect the numbers of participants with both SV95C and COA (4SC, NSAA, 6MWD) data at baseline. 4SC, Four-Stair Climb; 6MWD, Six-Minute Walk Distance; COA, clinical outcome assessment; NSAA, North Star Ambulatory Assessment; SV95C, Stride Velocity 95^th^ Centile.

At baseline, the correlations between SV95C and other motor function COAs were strong, with *r* values of 0.695 for 4SC velocity (*n* = 47), 0.611 for NSAA total score (*n* = 47), and 0.685 for 6MWD (*n* = 46) (Fig. 1).

**Fig. 2:**
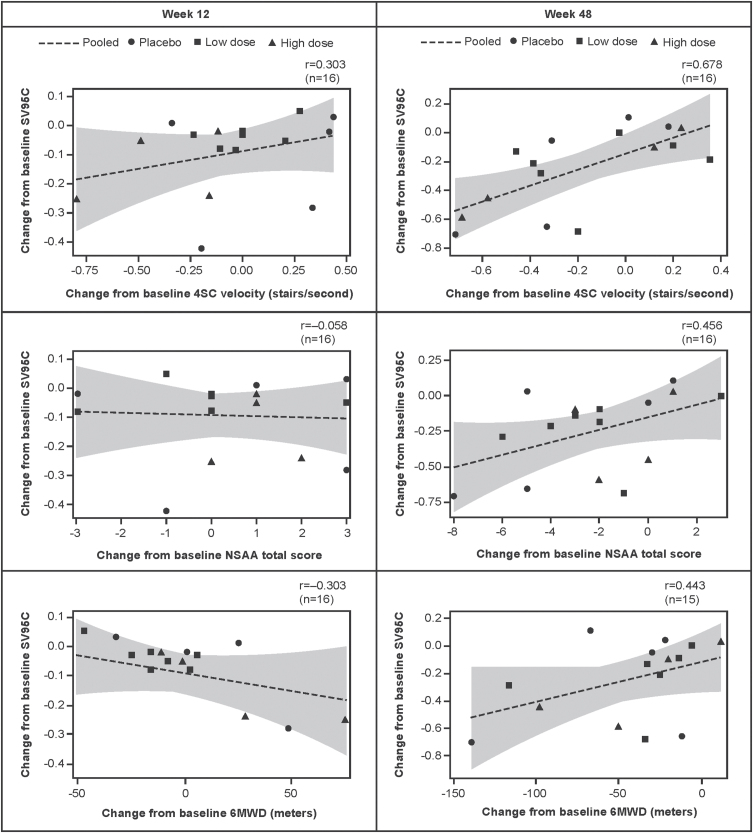
Associations between change from baseline in SV95C and change from baseline in 4SC velocity, NSAA total score, and 6MWD (regression lines with 95% confidence intervals for the predicted mean values). Pearson correlations. N’s reflect the numbers of participants with both SV95C and COA (4SC, NSAA, 6MWD) data at baseline and Week 12 (left panel) or Week 48 (right panel). 4SC, Four-Stair Climb; 6MWD, Six-Minute Walk Distance; COA, clinical outcome assessment; NSAA, North Star Ambulatory Assessment; SV95C, Stride Velocity 95^th^ Centile.

Correlations between change from baseline to Week 48 in SV95C and change from baseline to Week 48 in the other motor function COAs were moderate to strong, with *r* values of 0.678 for 4SC velocity (*n* = 16), 0.456 for NSAA (*n* = 16), and 0.443 for 6MWD (*n* = 15) (Fig. 2).

### Change from baseline in motor function COAs

**Fig. 3:**
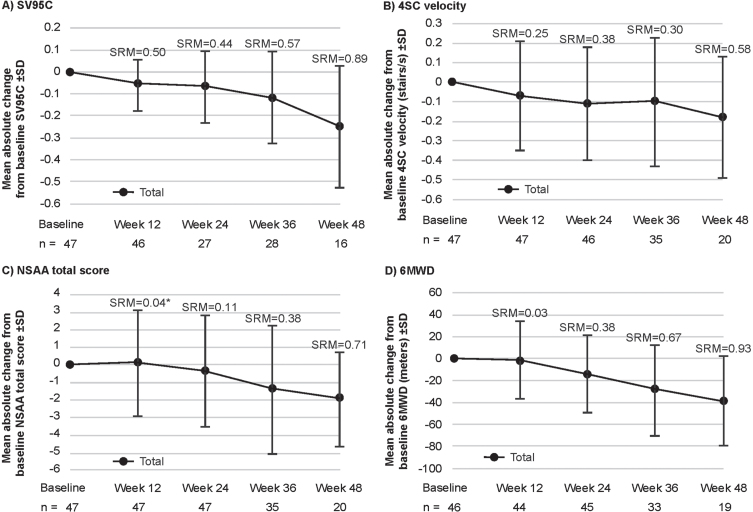
Mean absolute change from baseline in SV95C and other motor function COAs (pooled SV95C evaluable population: placebo and taldefgrobep alfa). ^*^Note that the SRM for NSAA at Week 12 represents an increase from baseline, while the longer-term data show a decrease. A: In the pooled SV95C evaluable population, mean absolute decline from baseline (±standard deviation) in SV95C (m/s) was –0.06±0.12 at Week 12 (*n* = 46), –0.07±0.16 at Week 24 (*n* = 27), –0.12±0.21 at Week 36 (*n* = 28), and –0.25±0.28 at Week 48 (*n* = 16). B: Mean absolute change from baseline in 4SC velocity (stairs per second) was –0.07±0.28 at Week 12 (*n* = 47), –0.11±0.29 at Week 24 (*n* = 46), –0.10±0.33 at Week 36 (*n* = 35), and –0.18±0.31 at Week 48 (*n* = 20). C: Mean absolute change from baseline in NSAA total score (points) was 0.13±3.03 at Week 12 (*n* = 47), –0.34±3.19 at Week 24 (*n* = 47), –1.40±3.67 at Week 36 (*n* = 35), and –1.90±2.69 at Week 48 (*n* = 20). D: Mean absolute change from baseline in 6MWD (meters; *n* = 44) was –1.09±36.29 at Week 12, –13.54±35.35 at Week 24 (*n* = 45), –27.87±41.51 at Week 36 (*n* = 33), and –38.48±41.33 at Week 48 (*n* = 19). 4SC, Four-Stair Climb; 6WMD, Six-Minute Walk Distance; COA, clinical outcome assessment; NSAA, North Star Ambulatory Assessment; SD, standard deviation; SRM, standardized response mean; SV95C, Stride Velocity 95^th^ Centile.

All motor function COAs assessed in this study (SV95C, 4SC velocity, NSAA total score, and 6MWD) declined over time. For the pooled SV95C evaluable population, mean absolute decline from baseline (±standard deviation) in SV95C (m/s) was –0.06±0.12 at Week 12 (*n* = 46), –0.07±0.16 at Week 24 (*n* = 27), –0.12±0.21 at Week 36 (*n* = 28), and –0.25±0.28 at Week 48 (*n* = 16) (Fig. 3A). Mean absolute changes for the other motor function COAs are shown in Fig. 3B–D. As results from analyses of absolute change split by taldefgrobep alfa versus placebo (Supplementary Figure 2 and Supplementary Figure 3) were generally comparable to those from the pooled analyses, pooled results are presented and discussed. Plots showing MMRM analyses of LSM change from baseline in motor function COAs are available in Supplementary Figure 4.

The SRMs for each COA at each timepoint are shown in Fig. 3. The SRMs at Week 12 were 0.50 for SV95C (*n* = 46), 0.25 for 4SC (*n* = 47), 0.04 for NSAA (*n* = 47), and 0.03 for 6MWD (*n* = 44) (Fig. 3). At Week 24, the SRMs were 0.44 for SV95C (*n* = 27), 0.38 for 4SC velocity (*n* = 46), 0.11 for NSAA (*n* = 47), and 0.38 for 6MWD (*n* = 45). At Week 36, the SRMs were 0.57 for SV95C (*n* = 28), 0.30 for 4SC velocity (*n* = 35), 0.38 for NSAA (*n* = 35), and 0.67 for 6MWD (*n* = 33). At Week 48, the SRMs were 0.89 for SV95C (*n* = 16), 0.58 for 4SC (*n* = 20), 0.71 for NSAA (*n* = 20), and 0.93 for 6MWD (*n* = 19).

## DISCUSSION

SV95C results from the Phase 2/3 SPITFIRE/WN40227 trial were consistent with those for the other motor function COAs used in the trial, which demonstrated a lack of drug effect [18]. Although the trial was discontinued due to futility and the early termination limited sample sizes at later timepoints [18], the assessment of SV95C generated novel insights that are of interest to the DMD community and may impact future development efforts in DMD. Key among these insights were the earlier detection of decline in ambulation using SV95C versus established clinic-based motor function COAs (based on the SRM analyses), the low variability of SV95C between patients (based on SD in mean absolute changes), and the consistency of SV95C with established motor function COAs (based on Pearson correlations between baseline values as well as changes over time).

Patient compliance with wearing and using the device during the prespecified study periods was generally good (Supplementary Figure 1). In its SV95C qualification opinion, the EMA defines the minimum data threshold for interpretation with acceptable variability as 50 hours and the optimal recording duration as 180 hours [10]. Approximately 10% (47/52) of patients were excluded from analyses because they did not meet this 50-hour threshold at baseline AND at least one post-baseline timepoint. Compliance (based on ≥50 recorded hours) was strongest at baseline (98.1% ; 51/52 patients) and weakest at Week 24 (51.9% ; 27/52 patients), and was between 82.4% (28/34 patients; Week 36) and 88.5% (46/52 patients; Week 12) at all other timepoints. Up to ∼600 hours were recorded from individual patients in a single 6-week period (recorded between visits every 3 months). These compliance data, while encouraging, should be considered in future study designs. The ActiMyo^®^ device has since been refined with a smaller and improved design (Syde is the new form factor currently available for use) [27], and this, in addition to SV95C’s recent qualification as a primary endpoint in pivotal DMD trials, will potentially contribute to improved compliance in the future. Overall, these findings demonstrate the feasibility of deploying wearable technology in international, multicenter pivotal trials.

Correlational analyses demonstrated the consistency of SV95C with established motor function COAs; these findings are in keeping with the significant correlations between SV95C (recorded over 180 hours at baseline) and the 6MWT, NSAA, and 4SC velocity at baseline reported in the EMA qualification opinion document [10]. Higher correlations between some traditional outcomes in DMD have been reported (e.g., *r* = 0.87 for time to stand from supine and 4SC and *r* = 0.75 for 6MWT and NSAA in 48 steroid-naïve patients with DMD [28]; *r* = 0.80 for NSAA and 10-meter Walk/Run in 61 patients with DMD [29]). It is important to note, however, that correlational analyses of traditional tests account for variability between the two assessments (typically measured on the same day), but not variability due to external factors like fatigue and attention on the day of the test. By contrast, a single measure of SV95C over a recording period accounts for the influence of these day-to-day factors. Analyses of absolute change in SV95C in SPITFIRE demonstrated its low variability despite the smaller sample size relative to the other motor function COAs.

Changes in SV95C over time observed in the SPITFIRE study were similar to the natural changes reported in a moderately larger sample of patients on a stable steroid regimen used to support the EMA primary qualification opinion [10]; these data showed mean m/s changes of –0.051 (0.113) at 12 weeks (*n* = 81), –0.080 (0.134) at 24 weeks (*n* = 59), –0.119 (0.186) at 36 weeks (*n* = 39), and –0.241 (0.234) at 48 weeks (*n* = 28).

The SRM effect sizes, while based on limited data, were similar to (and in most cases more modest than) those reported at similar timepoints in a larger population of patients with DMD in the EMA primary endpoint qualification opinion [10]. The SRM effect sizes calculated from the SPITFIRE study data suggested that SV95C was responsive to meaningful decline (based on prior estimates of clinically meaningful change, i.e., the minimal clinically important difference [MCID], or smallest change considered important to patients, clinicians, or significant others) at earlier timepoints than the NSAA, 4SC velocity, and 6MWD. Week 12 and Week 24 SRMs for SV95C exceeded those of the three other motor function COAs. These differences were particularly notable at the earliest timepoint assessed (Week 12)—at which point the SRM for SV95C already indicated moderate responsiveness to clinical change, and was double the SRM for 4SC, 12.5 times greater than the SRM for NSAA total score, and nearly 17 times greater than the SRM for 6MWD. By contrast, the SRMs of the other motor function COAs did not indicate moderate responsiveness until Week 36 (6MWD) or Week 48 (4SC velocity, NSAA total score). Notably, the SRM for NSAA at Week 12 shows an increase from baseline, while the longer-term data show a decrease.

At the later timepoints (Weeks 36 and 48), the SRMs for SV95C were surpassed only by those for 6MWD; both SV95C and 6MWD demonstrated moderate and large responsiveness at these respective timepoints. Thus, SV95C SRMs were higher than 4SC and NSAA total score SRMs at every timepoint measured in the study. These trends are consistent with data presented at the 2021 World Muscle Society congress, which demonstrated a significant change in SV95C at 3, 6, and 12 months, with respective SRMs of 0.45, 0.60, and 1.03 [30]. In contrast with the SV95C findings, the authors reported that significant changes in hospital-based assessments (6MWD and NSAA) were not detected until after 1 year.

To help interpret the changes observed in this study, it is important to consider previous estimates of clinically meaningful change. The MCID for SV95C was initially estimated in the EMA secondary qualification opinion document, using a distribution-based methodology (MCID = SD^*^√(1 — R)), as 0.1 m/s in a data set of 40 patients [31]. More recently, a shift in SV95C of 0.07 m/s has been reported as a meaningful change beyond measurement error for assessing improvement or decline, based on standard error of measurement distribution-based analyses [30]. Compared with the earlier MCID estimate, the SEM analyses were conducted using a larger but similar data set of 125 ambulant patients with DMD (aged 5–14 years) from six natural history studies and clinical trials [30, 32]. The study, which was presented at the 2021 World Muscle Society congress, also reported a minimal detectable change (MDC) of 0.13 m/s (80% confidence interval). Additionally, based on the results of distribution- and anchor-based analyses of meaningful change in SV95C, the EMA states in their primary qualification opinion that a change of 0.1 m/s in SV95C is a reasonable meaningful change threshold (MCT) estimate [10].

In our analyses of the pooled SV95C population from the SPITFIRE study, mean absolute change in SV95C had already reached 0.06 m/s by Week 12—the first post-baseline timepoint measured—and the SEM-based threshold of 0.07 m/s by Week 24. By Week 36, change in SV95C had exceeded the previously established MCID and MCT thresholds of 0.1 m/s [13]. By contrast, change in 6MWD did not reach published estimates of the MCID based on distribution-based methods (which range from 28.5 to 31.7 meters in broadly comparable populations [9, 10, 32]) until Week 48. Changes in NSAA and 4SC velocity did not reach estimated levels of clinically meaningful change (which range from 2.22–3.50 for NSAA and 0.29–0.47 for 4SC velocity in broadly comparable populations, based on both distribution- and anchor-based estimates [33–35]) at any timepoint measured in the study. Taken together, these findings highlight the potential enhanced sensitivity of SV95C, compared with established motor function COAs, for detecting meaningful decline in ambulation in patients with DMD.

When comparing the findings of the present study to previously published estimates of clinically meaningful change, it is important to consider differences in the populations in which these prior studies were conducted. Generally, the populations from which previous meaningful change estimates were derived were comparable to the SPITFIRE population evaluated (*N* = 52 ambulatory boys with DMD aged 6 to 11 years); however, some estimates (e.g., 6MWD) were derived from broader populations that included patients up to 20 years of age. The updated estimates of meaningful change in SV95C were based on 125 ambulant patients with DMD aged 5 to 14 years [30]. Estimates for 6MWD using distribution-based methods were derived from 174 ambulatory males aged 5 to 20 years [33]. For NSAA, one study using distribution-based methods provided estimates of meaningful change broken down by age range (<7 years = 2.66 points and 7 to 12 years = 2.80 points) [35]. Another study used distribution- and anchor-based estimates in a population 7 to 10 years old, which is similar to the SPITFIRE population (2.3–3.5 points, depending on the method used) [36]. An additional study used distribution- and anchor-based estimates in a population of 6- to 18-year-olds [34], which is similar to but broader than the SPITFIRE population. This latter population was also used to derive meaningful change estimates for 4SC velocity [34]. More recent studies estimating meaningful change on 4SC have provided estimates in seconds (0.7 MDC) [37] and speed (–0.034 MDC, –0.035 MCID using anchor-based methods) [38], which are not directly comparable to the velocity estimates derived here. In sum, the DMD populations on which meaningful change estimates have been based were similar to, but in some cases broader than, the SPITFIRE population assessed in this study; differences in study populations may have implications for the comparability of the results across studies.

SV95C also offers other benefits that are not available with traditional motor function COAs. Firstly, SV95C data are collected in the home environment, which reduces the burden of patient travel to study sites, lowers the threshold for study participation, and broadens the geographic reach of clinical trials. At the same time, compared with other endpoints (such as 6MWT) [9], SV95C may require fewer participants and shorter trial durations to achieve sufficient statistical power, which is especially advantageous in clinical trials of rare diseases. Additionally, while other assessments (6MWD, NSAA, and 4SC velocity) provide an important snapshot of a patient’s supposed maximal functional ability, continuous measurement of SV95C captures actual maximal performance [39], and is not influenced by motivation or fatigue on a given day. Finally, SV95C increases standardization by eliminating the reflex time of the physiotherapist who is recording timings. Although some traditional outcome measures, such as time to stand from supine [40] and the NSAA [41], have been successfully employed remotely, reducing patient burden and broadening the reach of clinical trials, these assessments still only capture patient abilities on the days they are administered (rather than continuously) and require active patient participation and scoring by a clinician. Therefore, the use of accurate and sensitive wearable sensors to passively measure patient motor function in a real-world setting may provide more realistic and objective assessments of performance.

Notably, while the traditional motor function COAs included in the SPITFIRE trial and compared with SV95C in the present study are common endpoints in DMD clinical trials and have been extensively validated in patients with DMD, none of the trials using these measures as primary endpoints have so far demonstrated drug efficacy and been used to support drug approval. The recent use of time to stand from supine velocity to support the approval of vamorolone for the treatment of DMD [40] and the demonstrated differences in this endpoint (and others) between untreated patients with DMD and patients treated with givinostat (Mercuri et al. *Lancet Neurology*, in press) highlight the importance of future comparisons of SV95C with other motor function COAs. Additionally, SV95C and time to stand from supine were among the secondary timed functional endpoints that demonstrated the consistent treatment benefit of ELEVIDYS in the recent EMBARK trial (which did not reach its primary endpoint, NSAA) [42], suggesting that these may be promising endpoints for detecting drug effects in clinical trials of DMD.

### Limitations

When studying a rare disease like DMD, the opportunity to generate data is often limited. One of the main limitations of this study was the small sample sizes, particularly for long-term SV95C data, and restricted patient numbers hindered a full interpretation of the longitudinal data. Importantly, the reduction in patient numbers across the trial period was not due to a lack of compliance but rather was primarily a result of the early termination of the SPITFIRE study; standard enrollment timelines were extended such that when the study was terminated, some patients were just beginning their participation in the study, while others had been participating for 24 to 48 weeks. Additionally, because SV95C was offered through a protocol amendment, only a subset of patients had the opportunity to participate in the SV95C sub-study. Data regarding the number of patients who were eligible for the sub-study but declined to participate are not available in the analysis database, which is a further limitation, as these data would be useful for understanding the overall feasibility of implementing SV95C in clinical trials. Given the lack of detailed information about participant corticosteroid regimens beyond the study requirements (receipt of prednisone, prednisolone, or deflazacort for at least 6 months pre-treatment, with no significant dose or regimen changes for at least 3 months pre-treatment and a stable regimen expected throughout the double-blind phase) as well as the age range and low participant numbers in this study, these SV95C data should not be considered natural history data. While results from the SPITFIRE study demonstrated the sensitivity of SV95C to disease changes in DMD, the lack of drug effect in the trial prevented evaluation of SV95C’s sensitivity to drug efficacy. Nevertheless, SV95C was among the secondary endpoints that demonstrated a treatment benefit in the EMBARK study of delandistrogene moxeparvovec (ELEVIDYS) in patients with DMD, although the study did not meet its primary endpoint (NSAA) [42]. Data included in the EMA qualification application also showed a positive change in SV95C following steroid initiation [10]. Comparisons with SV95C in the present study were also limited to the traditional motor function COAs used in the SPITFIRE trial, and SV95C was not compared with other important motor function COAs, such as time to stand from supine, which is one of the earliest affected milestones in DMD [43]. Furthermore, aside from walking and running episodes and climbing and descending stairs, data identifying specific activities that participants could perform were not available. This study was also not powered or designed to explore variability in SV95C based on the day of the week (e.g., weekday versus weekend) or time of year (e.g., winter versus summer), and the premature interruption of the study precluded seasonal analysis. The influence of local climate on SV95C is currently being investigated in a larger multisite study. Finally, this sub-population includes only patients who volunteered to wear the device, which may lead to overestimation of compliance.

## CONCLUSIONS

Although these findings should be confirmed in a larger population, the results suggest that SV95C may be more sensitive to detecting a decline in ambulation in DMD compared with established motor function COAs (6MWD, 4SC velocity, and NSAA), which declined at a slower rate. Using SV95C to detect early functional changes could have a significant impact on future trial design in DMD, as it has potential implications for the number of participants recruited and the duration of the trial.

In addition to potentially providing an earlier assessment of functional loss than established clinic-based motor function COAs, incorporating SV95C in clinical trials may positively impact study design by reducing sample size, study duration, patient burden, and the threshold to participate. Nonetheless, it remains important to currently consider SV95C as complementary to the in-clinic functional assessments.

The use of SV95C in the SPITFIRE study constitutes successful deployment of a wearable ankle device in a global pivotal study. The findings suggest the enhanced sensitivity of SV95C to a decline in the ambulation of patients with DMD compared with established motor function COAs, aligning with the data presented in the EMA qualification opinion [10]. SV95C has been qualified by the EMA as a primary endpoint in clinical trials of DMD to quantify the ambulatory ability of patients aged ≥4 years [10].

## Supplementary Material

Supplementary Material

## Data Availability

The data supporting the findings of this study are available on request from the corresponding author. The data are not publicly available due to privacy or ethical restrictions.
