# Supplementary material for: Stride Velocity 95th Centile Detects Decline in Ambulatory Function Over Shorter Intervals than the 6-Minute Walk Test or North Star Ambulatory Assessment in Duchenne Muscular Dystrophy
Source: J Neuromuscul Dis. 2024 Apr 30;11(3):701–14. doi: 10.3233/JND-230188 (PMC11091611; doi:10.3233/JND-230188)
Supplement: Supplementary Material [file jnd-11-jnd230188-s001.docx]

**Supplementary materials**

**Supplementary Figure 1. Summary of compliance: number of hours recorded by each patient at each visit, (intent-to-treat population)**

**
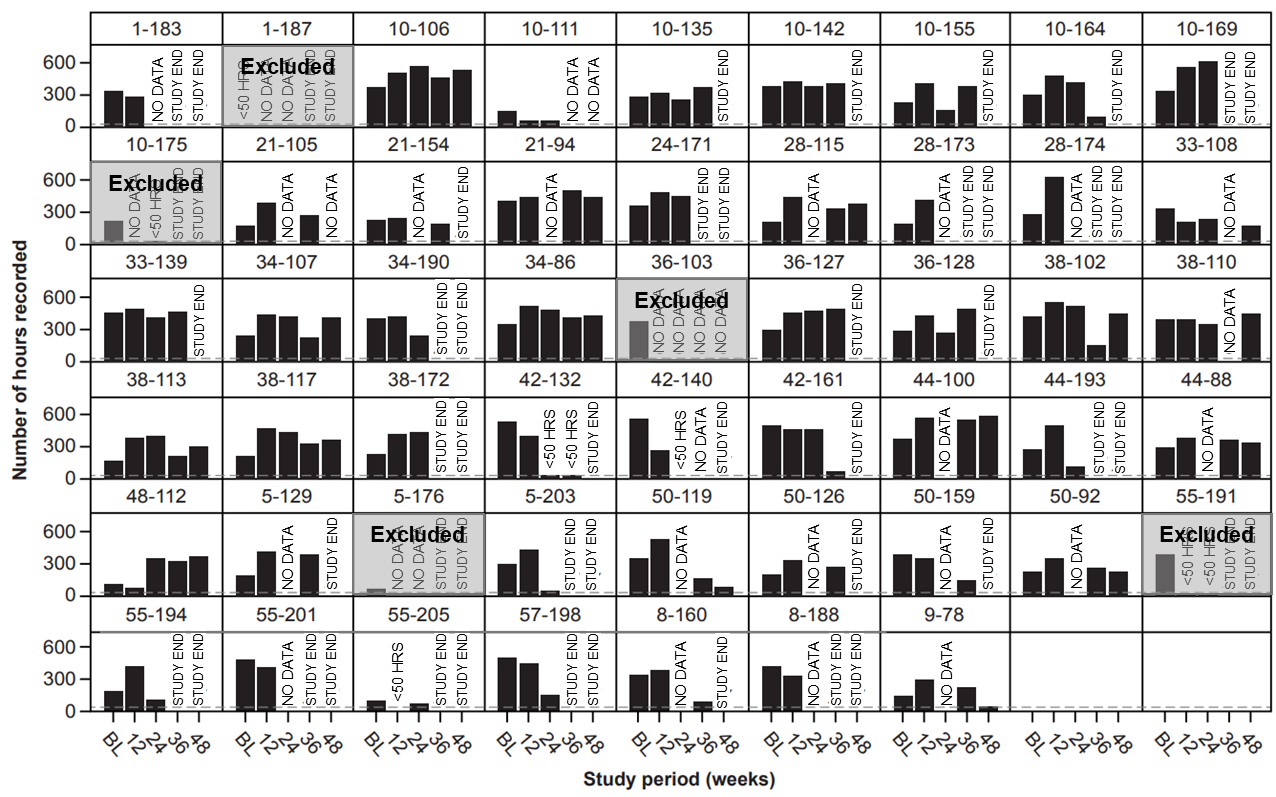
**

The dashed reference line denotes 50 hours, the minimum number of hours required for the assessment of stride velocity. Visits marked with ‘STUDY END’ are those for which the start of the data collection occurred after the decision to prematurely terminate the study was communicated (thus, no data collection would be expected and the lack of data is due to study termination rather than noncompliance). Visits denoted with ‘NO DATA’ are those for which the start of data collection occurred before study termination, and no hours were recorded. For visits marked with ‘<50 HRS,’ there were less than 50 but more than 0 hours recorded.

**Supplementary Figure 2. Mean ±SD absolute change from baseline in SV95C and other motor function COAs for placebo and pooled taldefgrobep alfa doses: SV95C evaluable population**


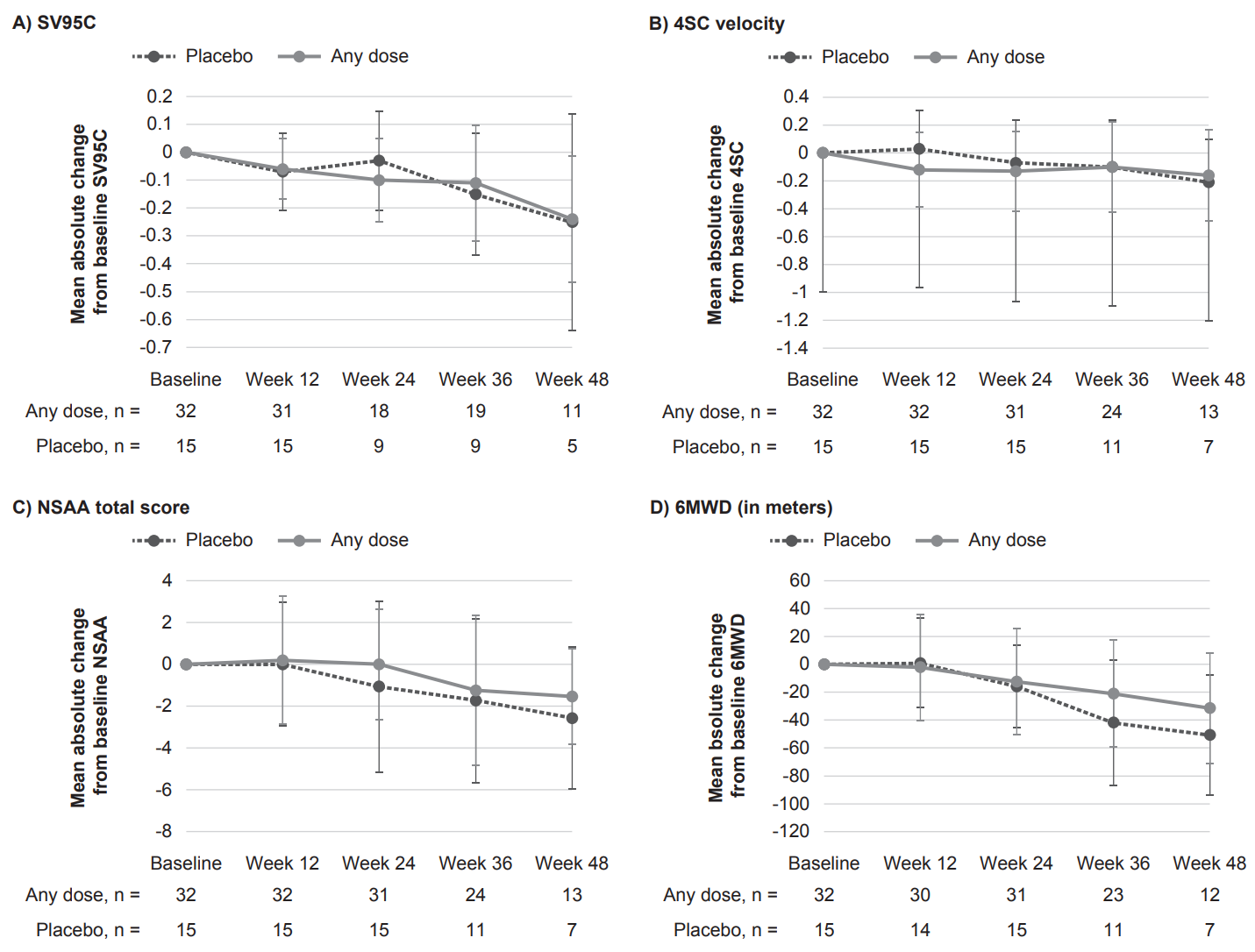
4SC, Four-Stair Climb; 6WMD, Six-Minute Walk Distance; COA, clinical outcome assessment; NSAA, North Star Ambulatory Assessment; SD, standard deviation; SV95C, Stride Velocity 95th Centile.

**Supplementary Figure 3. Mean ±SD absolute change from baseline in SV95C and other motor function COAs for placebo and low and high doses of taldefgrobep alfa**

**
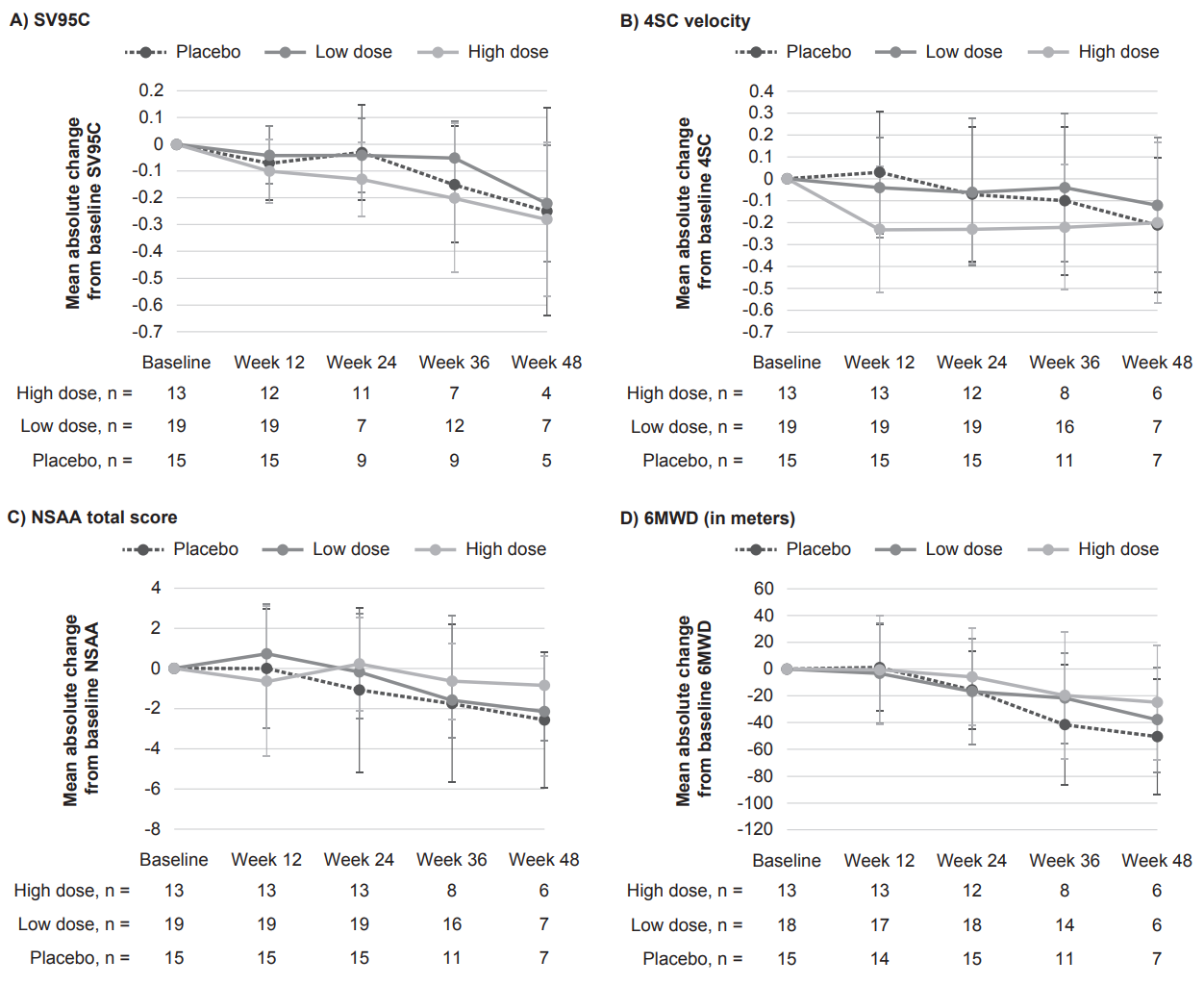
**

4SC, Four-Stair Climb; 6WMD, Six-Minute Walk Distance; COA, clinical outcome assessment; NSAA, North Star Ambulatory Assessment; SV95C, Stride Velocity 95th Centile.

**Supplementary Figure 4. LSM change from baseline in SV95C and other motor function COAs (MMRM, SV95C evaluable population)**


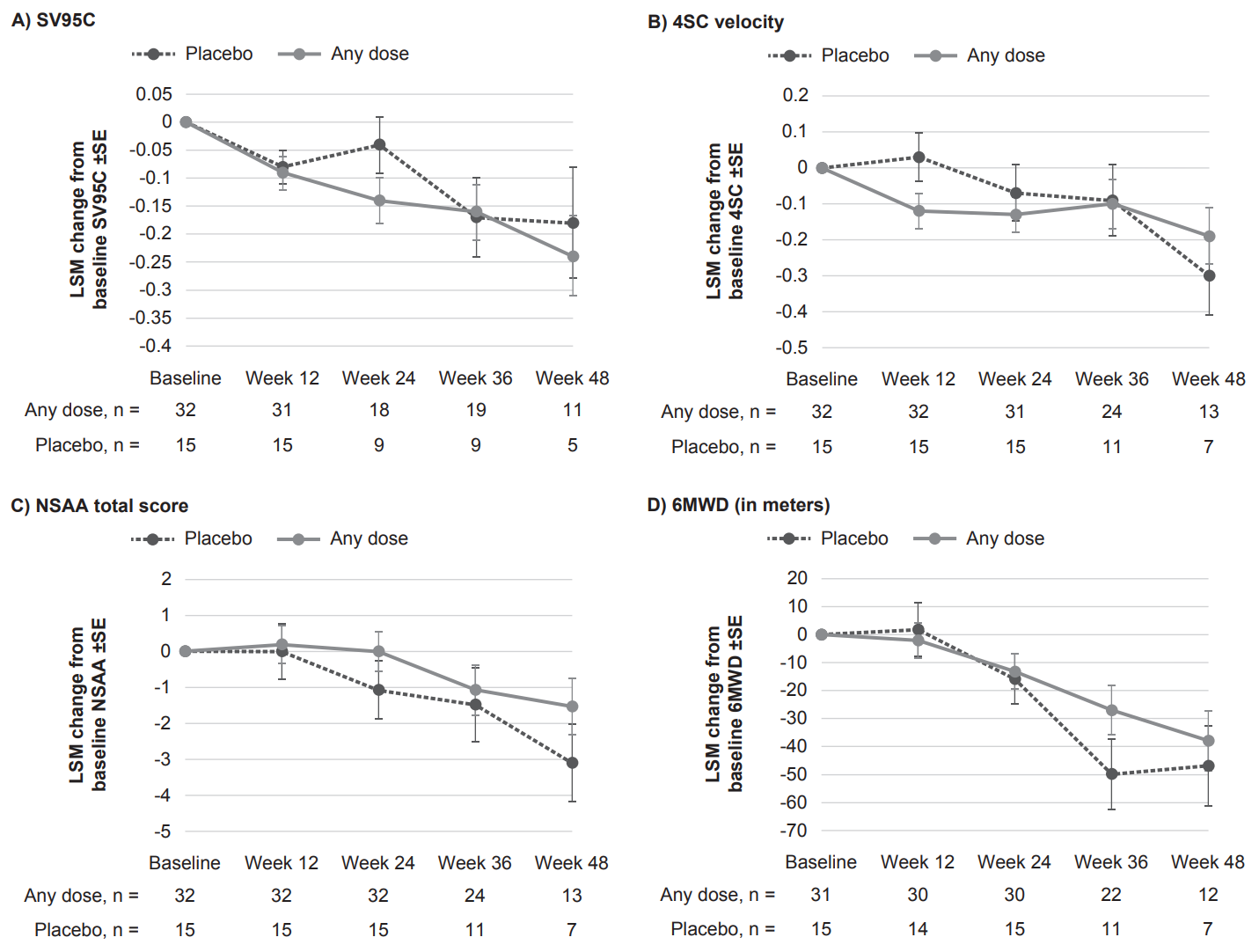
LSM change from baseline outcome measures was assessed via MMRM analyses. Fixed effects included in the model were treatment, visit, and treatment-by-visit interaction. Baseline score on the outcome measure (SV95C, 6MWD, 4SC velocity, or NSAA) was included as a covariate. Patient was entered as a random effect.

A) In the placebo group, LSM change (SE) from baseline in SV95C was –0.08 (0.03) at Week 12, –0.04 (0.05) at Week 24, –0.17 (0.07) at Week 36, and –0.18 (0.10) at Week 48. In the pooled dose group, LSM change (SE) from baseline in SV95C was –0.09 (0.03) at Week 12, –0.14 (0.04) at Week 24, –0.16 (0.05) at Week 36, and –0.24 (0.07) at Week 48. B) In the placebo group, LSM change (SE) from baseline in 4SC velocity was 0.03 (0.07) at Week 12, –0.07 (0.08) at Week 24, –0.09 (0.10) at Week 36, and –0.30 (0.11) at Week 48. In the pooled dose group, LSM change (SE) from baseline in 4SC velocity was –0.12 (0.05) at Week 12, –0.13 (0.05) at Week 24, –0.10 (0.07) at Week 36, and –0.19 (0.08) at Week 48. C) In the placebo group, LSM change (SE) from baseline in NSAA was 0.00 (0.79) at Week 12, –1.07 (0.82) at Week 24, –1.48 (1.04) at Week 36, and –3.10 (1.10) at Week 48. In the pooled dose group, LSM change (SE) from baseline in NSAA was 0.19 (0.54) at Week 12, 0.00 (0.56) at Week 24, –1.07 (0.71) at Week 36, and –1.53 (0.80) at Week 48. D) In the placebo group, LSM change (SE) from baseline in 6MWD was 1.8 (9.7) at Week 12, –15.8 (9.2) at Week 24, –49.8 (12.8) at Week 36, and –46.9 (14.5) at Week 48. In the pooled dose group, LSM change (SE) from baseline in 6MWD was –2.1 (6.6) at Week 12, –13.2 (6.5) at Week 24, –27.0 (9.1) at Week 36, and –37.9 (10.9) at Week 48.

4SC, Four-Stair Climb; 6WMD, Six-Minute Walk Distance; COA, clinical outcome assessment; LSM, least squares mean; MMRM, Mixed Model for Repeated Measures; NSAA, North Star Ambulatory Assessment; SE, standard error; SV95C, Stride Velocity 95th Centile.
